# Use of vortioxetine in treating obsessive-compulsive disorder: a case report

**DOI:** 10.1192/j.eurpsy.2024.1309

**Published:** 2024-08-27

**Authors:** B. Jiménez-Fernández, N. V. Motta-Rojas, X. Iborra-Vicheto, J. Cuevas-Esteban

**Affiliations:** Psychiatry, Hospital Universitari Germans Trias i Pujol, Badalona, Spain

## Abstract

**Introduction:**

Obsessive-compulsive disorder (OCD) is a chronic disorder with a wide range of manifestations but primarily intrusive thoughts (obsessions) and/or ritualized actions (compulsions) that can cause a huge distress in patients’ life. First-line treatment for OCD are selective serotonin reuptake inhibitors (SSRIs). Tricyclic antidepressants are used as second-line treatment due to secondary effects. Also antipsychotics such as aripiprazole are approved for treating OCD. Vortioxetine is has 5-HT3, 5-HT7 and 5-HT1D antagonists, 5-HT1B partial agonist and a 5-HT1A agonist and serotonin transporter inhibitor property. It is used in major depressive and anxiety disorders. A male 48 years old patient with an OCD diagnosis since he was 21, was reffered to psychiatry department. Previously, he had no response with SSRIs at full dosage and clomipramine 75mg was effective. At 46 years old, he had an acute myocardial infarction. He also admited not taking the medication regularly due to sexual disfunction and having affective symptoms related to the distress caused by OCD.

**Objectives:**

To evaluate efficacy of vortioxetine in treating OCD in a patient with contraindications for tricyclic antidepressants and no response to SSRIs.

**Methods:**

Clomipramine dose was reduced until discontinuation. After one week without treatment, basal scores for Hamilton Scale and Dimensional Yale-Brown Obsessive-Compulsive Scale (DY-BOCS) were collected. Same data was collected again after 10 weeks treatment.

**Results:**

The dosage of vortioxetine was progressively titrated until 20mg daily in 3 weeks lapse. Diazepam 5mg was added in case of insomnia or anxiety. Aripiprazole 5mg was added in the third week of treatment as adjunctive treatment due to the recurrence of some intrusive thoughts (discontinued by himself because of akathisia). Finally, the patient reported an improvement in affective and OCD symptoms in the sixth week of treatment that was sustained until the tenth week, when data was recollected. The patient did not refer sexual disfunction.

The pre and post results are summarized in tables 1 and 2.

**Table 1.** Hamilton Depresion Rating Scale (0-52)

Basal	Post 10-week treatment
21	4

Dimensional Y-BOCS (0-15)

**Table:** 

	Basal	Post 10-week treatment
Aggressive-related obsessions and compulsions	10	2
Religious-related obsessions and compulsions	5	1
Symmetry and order	7	1
Pollution and cleaning	0	0
Collecting and accumulation	2	0
Miscellaneous	10	3

**Conclusions:**

Vortioxetine might be a promising molecule for treating OCD in patients with contraindications for first and second-line treatments.

**Disclosure of Interest:**

None Declared

